# Evaluation of 3 molecular-based assays for microsatellite instability detection in formalin-fixed tissues of patients with endometrial and colorectal cancers

**DOI:** 10.1038/s41598-020-73421-5

**Published:** 2020-10-02

**Authors:** Pauline Gilson, Julien Levy, Marie Rouyer, Jessica Demange, Marie Husson, Céline Bonnet, Julia Salleron, Agnès Leroux, Jean-Louis Merlin, Alexandre Harlé

**Affiliations:** 1 Service de Biologie Moléculaire des Tumeurs, Institut de Cancérologie de Lorraine, CNRS UMR 7039 CRAN-Université de Lorraine, 6 avenue de Bourgogne CS 30519, 54519 Vandœuvre-lès-Nancy Cedex, France; 2 Département de Biopathologie, Institut de Cancérologie de Lorraine, 6 avenue de Bourgogne CS 30519, 54519 Vandœuvre-lès-Nancy Cedex, France; 3 Genetics Laboratory, CHRU Nancy, rue du Morvan, 54511 Vandœuvre-lès-Nancy, France; 4 Department of Biostatistics and Data Management, Institut de Cancérologie de Lorraine, 6 avenue de Bourgogne CS 30519, 54519 Vandœuvre-lès-Nancy Cedex, France

**Keywords:** Biological techniques, Cancer, Genetics, Molecular medicine, Oncology

## Abstract

Microsatellite instability (MSI) status is routinely assessed in patients with colorectal and endometrial cancers as it contributes to Lynch syndrome initial screening, tumour prognosis and selecting patients for immunotherapy. Currently, standard reference methods recommended for MSI/dMMR (deficient MisMatch Repair) testing consist of immunohistochemistry and pentaplex PCR-based assays, however, novel molecular-based techniques are emerging. Here, we aimed to evaluate the performance of a custom capture-based NGS method and the Bio-Rad ddPCR and Idylla approaches for the determination of MSI status for theranostic purposes in 30 formalin-fixed paraffin embedded (FFPE) tissue samples from patients with endometrial (n = 15) and colorectal (n = 15) cancers. All samples were previously characterised using IHC and Promega MSI Analysis System and these assays set as golden standard. Overall agreement, sensitivity and specificity of our custom-built NGS panel were 93.30%, 93.75% and 92.86% respectively. Overall agreement, sensitivity and specificity were 100% with the Idylla MSI system. The Bio-Rad ddPCR MSI assay showed a 100% concordance, sensitivity and specificity. The custom capture-based NGS, Bio-Rad ddPCR and Idylla approaches represent viable and complementary options to IHC and Promega MSI Analysis System for the detection of MSI. Bio-Rad ddPCR and Idylla MSI assays accounts for easy and fast screening assays while the NGS approach offers the advantages to simultaneously detect MSI and clinically relevant genomic alterations.

## Introduction

Microsatellites (MS) also termed simple sequence repeats (SRSs) or short tandem repeats (STRs) are composed of tandemly repeated DNA sequences of 1 to 6 nucleotides distributed throughout the genome^1^. MS are observed in both coding and non-coding regions and represent about 3% of the genome^2^. In normal tissues, DNA integrity and MS length are preserved due to a DNA mismatch repair (MMR) system that corrects DNA base mismatches made by DNA polymerase during the replication process or resulting from DNA damage^3^. MMR machinery consists of MutS complexes (MSH2/MSH6, MSH2/MSH3) that differentially recognize incorrect base pairing or insertion/deletion events and recruit MutL heterodimers (MLH1/PMS2, MLH1/PMS1 or MLH1/MLH3) to complete the DNA repair process^4^. In case of deficient MMR (dMMR) caused by genetic or epigenetic inactivating alterations in MMR genes (mainly *MLH1, MSH2, MSH6* and *PMS2*)^5^, genomic hypermutability arises and can predispose to cancer. Variance in microsatellite length then occurs within tumour DNA compared with matched-normal DNA, termed microsatellite instability (MSI).

MSI represents a molecular hallmark in an autosomal dominant inherited cancer predisposition syndrome known as Lynch syndrome (LS)^6^. MSI occurs in almost 95% of LS-associated malignancies, mainly colorectal and endometrial cancers and less frequently tumours of the small intestine, stomach, biliary system, ovarium, brain, upper urinary tract and skin^7^. In addition to familial conditions, MSI is also observed in sporadic cancers (2/3 of MSI cases) and is frequently associated with hypermethylation of the *MLH1* gene^5^. A study conducted on 12,019 cancer samples across 32 different tumour subtypes concluded that more than 2% of these cancers across 24 tumour subtypes exhibited MSI, including 17% of endometrial carcinomas, 9% of gastric adenocarcinomas and 6% of colorectal adenocarcinomas^8^.

Identification of MMR-deficient tumours is critical for therapeutic choice and clinical decisions. MSI or MMR statuses are tested in daily practice for the initial screening of Lynch syndrome in context of early age at onset of colorectal and endometrial cancers and/or informative personal or family medical histories^9^. Since few years, some expert guidelines even recommend an universal screening for all patients with newly diagnosed colorectal cancers in order to increase the identification of Lynch syndrome cases^10,11^. Most but not all studies indicated that MSI is associated with improved outcomes in metastatic and non-metastatic colorectal cancers^12–15^. Based on these data, adjuvant treatment decision in stage-II colorectal cancers is made depending on MSI status, according to the National Comprehensive Cancer Network (NCCN) guidelines^16^. Correlation between MSI status and prognosis is still controversial in endometrial cancers however several studies also suggested a more favourable prognosis in MSI molecular subtype^17,18^. MSI phenotype also contributes to treatment personalization since the presence of MSI in colorectal tumours predicted poor response to carboplatin, cisplatin or fluorouracil-based adjuvant chemotherapy while an improved response to oxaliplatin and irinotecan was observed^19–21^. Since 2017, MSI represents a valuable agnostic biomarker to predict response to immune checkpoint inhibitors (ICI) in several solid tumours regardless of their primary origin^22,23^. MSI is highly correlated with hypermutated, neoantigen-rich tumours and an active immune microenvironment that may explain the observed high efficacy of ICI^24–26^. Of note, Le et al*.* revealed an objective response rate to pembrolizumab of 52% and 53% in MSI colorectal and endometrial cancers respectively^8^.

Immunohistochemistry (IHC) (evaluation of MMR protein expression including MLH1, MSH2, MSH6 and PMS2) and pentaplex PCR-based assays (directed against 5 microsatellite regions including at least *BAT-25* and *BAT-26* mononucleotide markers such as Promega MSI Analysis System) are currently the reference assays recommended for dMMR/MSI testing^9,27–29^. However, novel molecular-based methods emerge for the detection of MSI, including different real-time PCR and droplet digital PCR (ddPCR)-based methods^30–33^ as well as various custom Next-Generation Sequencing (NGS) approaches^34–43^. In this study, we aimed to evaluate the performance of 3 molecular assays, including a custom capture-based NGS approach, Idylla MSI and Bio-Rad ddPCR MSI assays, for the determination of MSI status for theranostic purposes in patients with endometrial and colorectal cancers.

## Results

### Evaluation of the custom capture-based NGS, Idylla MSI and Bio-Rad ddPCR MSI assays compared to the standard reference methods

The custom-made NGS panel and the Bio-Rad ddPCR MSI assay yielded valid results for all the samples, including those with intermediate (E1, E3–E4, E6–E15, C16, C18–C23, C26–C30) and low DNA quality (E2, E5 and C17 samples). The mutations detected in the samples by our custom NGS panel were detailed in Supplementary Table S1 online. The Idylla MSI assay gave an invalid result for 1 out of 30 samples (E8 with intermediate DNA quality) (i.e. 3.3% of invalid results, 95% CI [0.1%; 17.2%]). Results from 12 out of 15 endometrial cancer samples (E1–E7, E11–E15) and 13 out of 15 colorectal cancer samples (C16–C23, C25–C26, C28–C30) were concordant between the custom NGS approach, the Idylla MSI assay, the Bio-Rad ddPCR MSI assay and both standard reference methods (IHC and Promega MSI Analysis System) (Table 1). Samples (E10, C24) that gave doubtful results from IHC testing obtained concordant results between the custom capture-based NGS approach, the Idylla MSI assay, the Bio-Rad ddPCR MSI assay and the gold-standard Promega MSI Analysis System.

**Table 1:** MMR/MSI status obtained by custom capture-based NGS, Idylla MSI assay, Bio-Rad ddPCR MSI assay, IHC and Promega MSI Analysis System.

Sample ID	DNA quality ($\Delta$Cq)	IHC (MMR proteins whose expression was lost)	Promega MSI Analysis System (number of markers out of 5 altered)	Idylla MSI assay (number of markers out of 7 altered)	Custom capture-based NGS (run-specific/global overall distance scores)	Bio-Rad ddPCR MSI assay
E1	0.8	dMMR (MLH1, PMS2)	MSI (5/5)	MSI-HC (7/7)	MSI-LC (8.1/5.5)	MSI
E2	7.0	dMMR (MLH1, PMS2)	MSI (4/5)	MSI-HC (4/7)	MSI-LC (13.2/12.8)	MSI
E3	1.3	dMMR (MLH1, PMS2)	MSI (5/5)	MSI-HC (4/7)	MSI-LC (9/8.6)	MSI
E4	1.8	dMMR (MLH1, PMS2)	N/A	MSI-HC (4/7)	MSI-LC (9.1/8)	MSI
E5	7.0	dMMR (MSH2, MSH6)	MSI (5/5)	MSI-HC (4/7)	MSI-HC (23.9/22.9)	MSI
E6	3.1	dMMR (MSH2, MSH6)	MSI (5/5)	MSI-HC (6/7)	MSI-HC (20.3/19.3)	MSI
E7	4.0	dMMR (MSH6)	MSI (5/5)	MSI-HC (4/7)	MSI-LC (8.8/7.8)	MSI
E8	4.1	dMMR (MSH2)	MSI (5/5)	Invalid^a^	MSS (1.6/1.2)	MSI
E9	2.8	dMMR (PMS2)	MSS (0/5)	MSS (0/7)	MSS (1.1/1.6)	MSS
E10	4.0	Doubtful for MSH6 protein	MSS (0/5)	MSS (0/7)	MSS (1.7/2.1)	MSS
E11	1.6	pMMR	MSS (0/5)	MSS (0/7)	MSS (1.7/2.8)	MSS
E12	3.9	pMMR	MSS (0/5)	MSS (0/7)	MSS (0.8/1.3)	MSS
E13	2.5	pMMR	MSS (0/5)	MSS (0/7)	MSS (1.2/1.4)	MSS
E14	3.5	pMMR	MSS (0/5)	MSS (0/7)	MSS (1.7/2.5)	MSS
E15	3.4	pMMR	MSS (0/5)	MSS (0/7)	MSS (2/1.5)	MSS
C16	1.5	dMMR (MLH1, MSH2, MSH6, PMS2)	MSI (5/5)	MSI-HC (5/7)	MSI-HC (26.7/25.7)	MSI
C17	9.8	dMMR (MLH1, PMS2)	MSI (2/5)	MSI-HC (6/7)	MSI-LC (6.7/5)	MSI
C18	1.5	dMMR (MLH1, PMS2)	MSI(5/5)	MSI-HC (7/7)	MSI-HC (23.4/22.6)	MSI
C19	1.8	dMMR (MLH1, PMS2)	MSI (5/5)	MSI-HC (5/7)	MSI-HC (15.7/14.7)	MSI
C20	4.0	dMMR (MLH1, PMS2)	MSI (5/5)	MSI-HC (6/7)	MSI-HC (26.8/26.3)	MSI
C21	2.2	dMMR (MSH2, MSH6)	MSI (5/5)	MSI-HC (7/7)	MSI-HC (31.6/29.1)	MSI
C22	3.2	dMMR (PMS2)	MSI (5/5)	MSI-HC (6/7)	MSI-HC (26.3/23.8)	MSI
C23	3.9	N/A	MSI (5/5)	MSI-HC (7/7)	MSI-HC (15.7/14.6)	MSI
C24	− 0.1	Doubtful for MLH1 protein	MSS (0/5)	MSS (0/7)	MSS (1.2/1.7)	MSS
C25	− 0.1	pMMR	MSS (0/5)	MSS (0/7)	MSS (1.3/2.2)	MSS
C26	4.3	pMMR	MSS (0/5)	MSS (0/7)	MSS (1/2.2)	MSS
C27	3.4	pMMR	MSS (0/5)	MSS (0/7)	MSI-LC (5.3/6.8)	MSS
C28	2.0	pMMR	MSS (0/5)	MSS (0/7)	MSS (1.6/2.1)	MSS
C29	1.1	pMMR	MSS (0/5)	MSS (0/7)	MSS (0.9/2.2)	MSS
C30	5.5	pMMR	MSS (0/5)	MSS (0/7)	MSS (1.9/3.4)	MSS

For each sample, DNA quality was determined by a qPCR-based quality control assay and Delta-Cq ($\Delta$Cq) were calculated as follows: Cq value of the sample − Cq value of the internal control included in the kit. Samples with $\Delta$Cq ≤ 0 are characterized by a high DNA quality, those with $\Delta$Cq between 0 and 6 are of intermediate DNA quality, and those with $\Delta$Cq ≥ 6 are of poor DNA quality.

dMMR: deficient MMR; MSI: microsatellite instabilitye; MSI-HC: microsatellite instability with high confidence, MSI-LC: microsatellite instability with low confidence; MSS: microsatellite stability, N/A: not available; pMMR: proficient MMR.

^a^2 markers not correctly amplified.

### Analysis of discrepant results

Three samples (E8, E9, C27), that showed intermediate DNA quality ($\Delta$Cq of 4.1, 2.8, 3.4 respectively), displayed discordant results. All MS molecular approaches identified the E9 endometrial cancer sample as MSS while sole IHC testing showed a MSI/dMMR phenotype (with a loss of expression of the PMS2 protein). Concerning the MMR/MSI screening in the E8 endometrial cancer sample, the Idylla platform showed an invalid result, the custom NGS approach identified a MSS phenotype and the other techniques found an MSI phenotype (see Supplementary Table S2 online). Finally, the C27 colorectal cancer sample was shown as MSS by all approaches except for custom capture-based NGS that identified MSI with low confidence in the sample (see Supplementary Table S2 online).

### Overall agreement, sensitivity and specificity of the custom NGS, Idylla MSI and Bio-Rad ddPCR MSI assays

Considering both IHC and Promega MSI Analysis System as the gold standard, the overall agreement, sensitivity and specificity of the custom NGS approach were 93.30%, 93.75% and 92.86% respectively (Table 2). A 100% concordance, sensitivity and specificity were reached with the Idylla platform compared to standard reference methods. The Bio-Rad ddPCR MSI assay had an overall agreement, a sensitivity and a specificity of 100%.

**Table 2:** Overall agreement, sensitivity and specificity of the custom capture-based NGS approach, the Idylla MSI assay and the Bio-Rad ddPCR MSI assay, using both IHC and Promega MSI Analysis System as the gold standard.

	Custom capture-based NGS	Idylla MSI assay	Bio-Rad ddPCR MSI assay
Overall agreement, % (N)	93.30% [77.9%;99.2%] (28/30)	100% [88.1%;100%] (29/29^a^)	100% [88.4%;100%] (30/30)
Sensitivity, % (N)	93.75% [69.7%;99.8%] (15/16)	100% [78.2%;100%] (15/15)	100% [79.4%;100%] (16/16)
Specificity, % (N)	92.86% [66.1%;99.8%] (13/14)	100% [76.8%;100%] (14/14)	100% [76.8%;100%] (14/14)

Results from Promega MSI Analysis System were used as the reference for cases with equivocal IHC data.

Results are expressed as percentage with 95% confidence interval, and the number of samples used to calculate the overall agreement, sensitivity and specificity.

^a^One invalid result.

## Discussion

Determining MMR/MSI status is becoming crucial in endometrial and colorectal cancers as it contributes to hereditary screening and it greatly helps for cancer prognosis stratification and treatment choices. MMR protein IHC staining and Promega MSI five-marker-based detection system are considered as reliable techniques recommended for the determination of dMMR/MSI, however novel molecular-based approaches have recently been introduced. Herein, the clinical performance of a custom capture-based NGS approach, the Idylla MSI assay and the Bio-Rad ddPCR MSI assay have been evaluated for theranostic purposes in 15 endometrial and 15 colorectal cancer samples (all stages) in comparison with the standard reference methods.

Overall agreement of the custom capture-based NGS panel, the Idylla MSI system and the Bio-Rad ddPCR MSI assay was 93.30%, 100% and 100% respectively. Based on these data, results from the 3 molecular-based methods showed a high agreement with the current standard-of-care tests, hence representing ancillary options for MSI assessment in routine practice. These methods are shown applicable in both endometrial and colorectal cancer FFPE samples as well as in samples with low DNA quality (E2, E5, C17). Interestingly, all gave conclusive results and showed a perfect concordance with the pentaplex MSI PCR assay in the case of equivocal IHC results (E10, C24) suggesting that these approaches can retrieve samples that don’t reach conditions for IHC. In the case of E9 sample displaying discordant results between IHC and Promega system reference methods (i.e. isolated loss of PMS2 protein but MSS), the custom NGS, the Idylla and the Bio-Rad ddPCR approaches allowed to confirm the MSS status. Considering the crucial role of MSI as a predictive marker of immunotherapy efficacy, this illustrates the need to combine both IHC and molecular-based assays for MMR/MSI testing prior to immunotherapy initiation, in order to reduce the chances of misdiagnosis and subsequent resistance to treatment, as previously suggested by recent studies^29,44^.

Despite a high concordance, discrepant results among molecular methods were observed in 2 out of the 30 cases (E8, C27). Focusing on the E8 sample, the discordant results and the invalidity of the Idylla MSI assay could be related in part to the long-term storage of the FFPE blocks as the standard reference methods and the evaluated approaches were performed more than 2 years and 6 years after tumour sampling respectively (see Supplementary Table S3 online). However, the analysis of the DNA extracted from the E8 sample showed a fairly well-preserved DNA ($\Delta$Cq = 4.1) at the time of molecular analyses (see Supplementary Table S2 online). The custom NGS approach gave discordant results with other molecular techniques (i.e. MSI-LC while MSS with other approaches) for the C27 sample. The analysis of run-specific and global overall distance scores provided by the MSI calculation algorithm actually revealed equivocal results (run-specific overall score < 6 while global overall score > 6), highlighting in such cases the limit of this approach and the need to confirm the results of MSI-LC by a complementary technique (see Supplementary Table S2 online).

IHC and MSI molecular methods are complementary to each other in nature. IHC identifies the defective MMR proteins responsible for MSI phenotype while molecular methods detect the functional consequence of mismatch repair deficiency regardless of the mechanism involved^45^. Hence, IHC is the sole methodology able to guide for MMR genes to investigate for damaging alterations. In return, IHC can yield false-negative results due to rare but not negligible cases of missense mutations in MMR genes contributing to non-functional but antigenically intact protein^46,47^ that would be identified by molecular testing.

The fully-automated Idylla platform offers MSI results in less than 2.5 h without requiring preliminary DNA isolation and with a minimal hands-on-time. Data interpretation is also simplified with the generation of automated reports that make it easily implementable in all clinical laboratories. Several studies already evaluated the Idylla system for MSI determination in colorectal cancers and showed equivalent concordance, sensitivity and specificity to our study associated with a low rate of invalid results^31–33,48^. Interestingly, we showed here similar performance in endometrial cancers than those observed in colorectal cancers.

With a 100% sensitivity and specificity, the Bio-Rad ddPCR MSI assay consists in a fast and cost-effective multiplex assay compatible with large-scale testing of patients, though requiring a specialized laboratory to interpret the data. In a recent study, a custom implementation of ddPCR using specialized targets allowed to reach an unmatched sensitivity for MSI screening compared to previously used approaches, making this methodology applicable for MSI analysis in liquid biopsies^30^.

In this study, our custom NGS panel was associated with a 93.75% sensitivity and a 92.86% specificity. This method offers the advantage over other tested methods to simultaneously detect actionable genomic alterations in cancer-related genes that predict response to targeted therapies. This all-in-one strategy is particularly relevant for metastatic colorectal cancer cases whose MSI status contributes to tumour prognosis and patient selection for immunotherapy while KRAS, NRAS and BRAF genotyping helps for targeted therapies decision. Conversely, this technically challenging strategy requires stringent quality of DNA and is significantly more time-consuming and expensive, making its use limited to cancer cases that need both MSI determination and genomic profiling. In this study, we chose a moderate throughput approach, but broader approaches are described in the literature. Some NGS panels provide, in addition to the MSI status and the genomic profile, the determination of the tumour mutation burden (TMB) score, a complementary predictive tumour biomarker of immunotherapy efficacy^36,49^. Notably, The Food and Drug Administration (FDA) recently approved a NGS-based broad companion diagnostic (FoundationOne CDx (F1CDx) from Foundation Medicine) for the simultaneous analysis of MSI status, genomic aberrations and TMB in all solid tumors. Those imply more complex methods of sequencing and analysis and subsequently a higher cost.

In conclusion, the custom NGS, Idylla and Bio-Rad ddPCR MSI testing methods could effectively surrogate all current standard-of-care assays routinely performed on colorectal and endometrial cancers. They have different advantages and limitations that have to be considered in order to choose the most appropriate approach depending on the clinical and biological context.

## Materials and methods

### Sample selection

**Figure 1:**
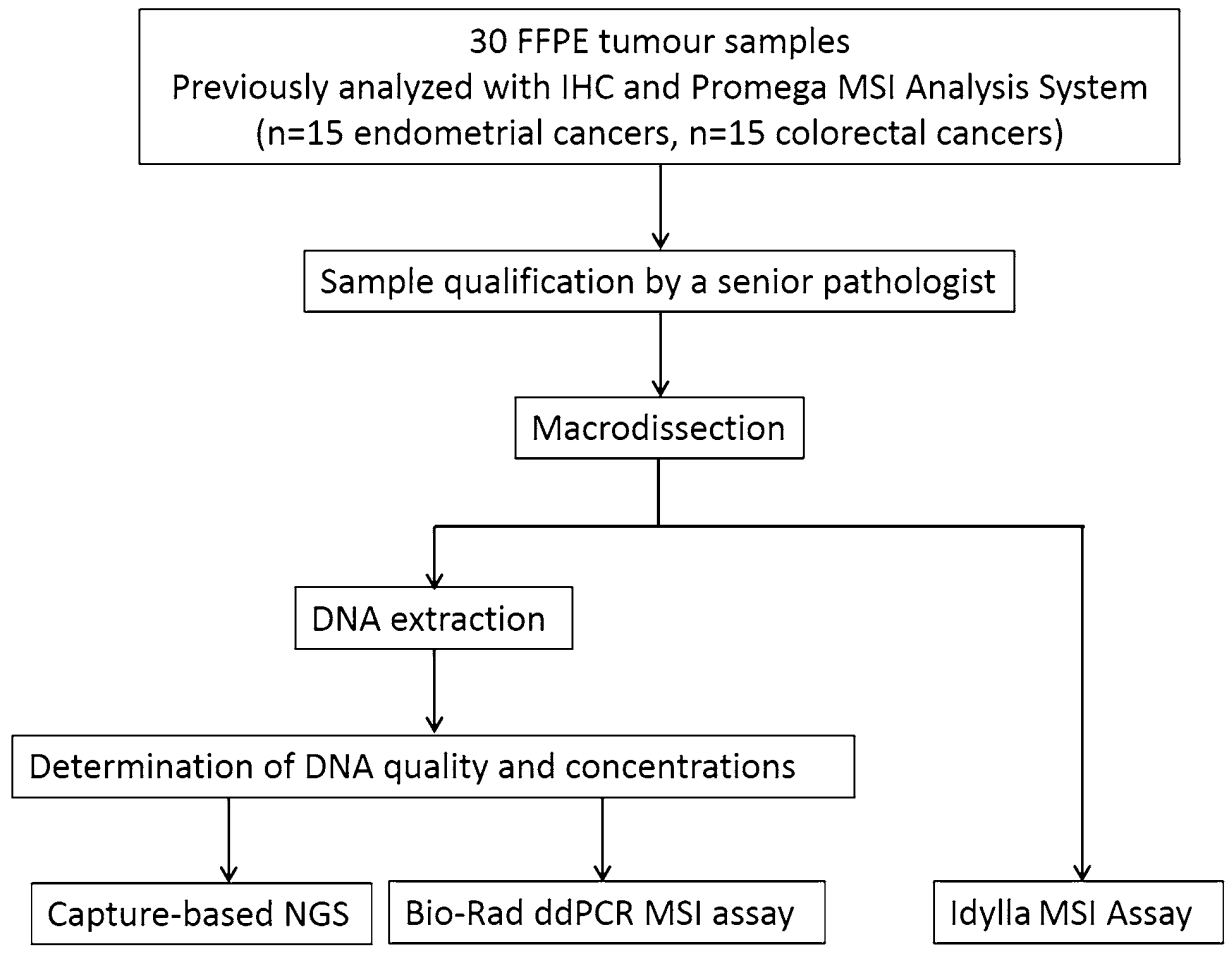
Study workflow. ddPCR: droplet digital PCR; FFPE: Formalin-Fixed Paraffin-Embedded; IHC: ImmunoHistoChemistry, MSI: Microsatellite Instability; NGS: Next Generation Sequencing.

Thirty formalin-fixed paraffin-embedded (FFPE) tumour samples from patients with endometrial or colorectal cancers were retrospectively selected among the biological samples collection of Institut de Cancérologie de Lorraine (ICL, Nancy, France) and Centre Hospitalier Régional Universitaire de Nancy (CHRU, Nancy, France). All samples were fixed with 10% neutral phosphate-buffered formalin (NBF) within 1 h from tissue harvesting. The duration of fixation varied depending on the size of the biological material. For all specimens, the total fixation time ranged from 8 to 48 h. These samples with at least 20% tumour cell content were collected between October 2013 and January 2020, once the determination of MSI status was obtained by standard routine care (IHC first and confirmation by pentaplex PCR-based assay) for patient cancer management (Fig. 1). The duration between the time of analysis and tumour sampling is detailed in Supplementary Table S3 online. All patients included in this study provided written informed consent for the analysis of MSI status. Approval from the ethical and scientific board of Institut de Cancérologie de Lorraine was granted for this study. All experiments and methods were performed in accordance with the relevant guidelines and regulations. Data were anonymized prior to use for the study. Data from custom NGS, Idylla and ddPCR approaches were analyzed by an experienced biologist who was blinded to the MSI/dMMR results obtained by routine care.

Among the samples selected, 15 were from patients with colorectal cancers and 15 from patients with endometrial cancers. The characteristics of the selected patients are presented in Table 3.

**Table 3:** Characteristics of the 30 selected patients.

	Colorectal cancers (N = 15)	Endometrial cancers (N = 15)
**Gender**		
Female	9	15
Male	7	
**Age at diagnosis (years)**		
Median [interquartile range]	67 [58–70]	64 [55–69]
Histological subtype (according to WHO classification)	Well differentiated: 5	Endometrioid grade 1: 3
	Moderate differentiated: 9	Endometrioid grade 2: 9
	Poor to moderate differentiated: 1	Endometrioid grade 3: 3

### Tumour tissue macrodissection and DNA extraction

FFPE tissues were macrodissected after hematoxylin–eosin slide examination and determination of tumour content by an experienced pathologist.

For NGS and ddPCR assays, DNA was extracted using the QIAamp Generead DNA FFPE tissue kit (Qiagen, Hilden, Germany) and DNA concentrations were measured using the Qubit dsDNA HS assay kit and Qubit 3.0 Fluorometer instrument according to the manufacturer's recommendations (ThermoFisher Scientific, Courtaboeuf, France).

For Promega MSI Analysis System, DNA was extracted using the NucleoSpin DNA FFPE XS kit (Macherey–Nagel, Hoerdt, France) and DNA were quantified by spectrophotometry using NanoDrop instrument (ThermoFisher Scientific).

DNA quality was determined by a qPCR-based quality control assay using the TruSeq FFPE DNA Library Prep QC kit (Illumina, San Diego, USA) and the LightCycler 480 Software W UDF 2.0.0 (Roche Diagnostics, Meylan, France). For each sample, Delta-Cq ($\Delta$Cq) were calculated as follows: Cq value of the sample − Cq value of the internal control included in the kit. Samples with $\Delta$Cq ≤ 0 are characterized by a high DNA quality, those with $\Delta$Cq between 0 and 6 are of intermediate DNA quality, and those with $\Delta$Cq ≥ 6 are of poor DNA quality.

### Promega MSI Analysis System version 1.2

The MSI Analysis System (Promega, Charbonnières-les-Bains, France) is designed for the analysis of 5 monomorphic mononucleotide repeat markers (*BAT-25, BAT-26, MONO-27, NR-21* and *NR-24*) for MSI typing and two highly polymorphic pentanucleotide repeat markers (*Penta C* and *Penta D*) for sample identification. The assays were performed on tumour samples using 50 ng of extracted DNA input. MSI, MSS and blank controls were used in each run. PCR was performed on the Applied Biosystems Veriti thermal cycler instrument (ThermoFisher Scientific). Once amplified, samples were analyzed by capillary electrophoresis using the 3130 genetic analyzer (Applied Biosystems, Thermo Fisher Scientific, Inc.). Data were treated with GeneMapper Software v4.1 (ThermoFisher Scientific). Tumours were considered MSI-H if at least 2 markers out of 5 (≥ 40% of MS markers) were unstable.

### Immunohistochemistry of MMR proteins

Immunohistochemistry staining of MMR proteins was performed on 5 µm-thick FFPE tumour tissue sections on a BenchMark ULTRA instrument (Ventana Medical Systems Inc., Tucson, AZ) with OptiView DAB IHC Detection Kit (Ventana) and the following antibodies: MHL1 (clone M1, Ventana), MSH2 (clone G219-1129, Ventana), MSH6 (clone 44, Ventana) and PMS2 (clone EPR3947, Cell Marque, Rocklin CA, USA). An absence of nuclear staining within the tumour identified the loss of expression of the targeted MMR protein (dMMR). For each antibody, staining pattern was checked by a senior pathologist using positive internal or external controls.

### Biological characteristics of the selected samples

All samples were retrospectively chosen once MMR/MSI status was determined by gold standard IHC and pentaplex PCR-based assays during routine clinical care (Table 4). Tumour content for the 15 endometrial cancer samples (E1–E15) ranged from 20 to 90% while DNA concentrations were between 6.8 to 72.9 ng/µL. Eight out of the 15 were defined as having dMMR/MSI (E1–E8). IHC testing showed a loss of expression of MLH1 and PMS2 proteins in 4 samples (E1–E4), a loss of MS2 and MSH6 proteins in 2 samples (E5–E6), an altered expression of MSH6 protein in 1 sample (E7) and an altered expression of MSH2 protein in 1 sample (E8). Five out of the 15 endometrial cancer samples were considered pMMR (proficient MMR)/MSS based on the IHC and pentaplex PCR results (E11–E15). IHC results from 1 sample (E10) were non-contributory (due to a low and heterogeneous expression of MSH6 protein) while the PCR-based assay identified a MSS phenotype. Finally, discrepant results between IHC (dMMR with loss of PMS2 expression) and pentaplex PCR-based assay (MSS) were obtained for 1 sample (E9).

**Table 4:** Biological characteristics of the samples.

Tumour origin	Sample ID	Tumour content ( %)	Sampling date ( year)	DNA concentrations ( ng/µL)	MMR status by IHC (MMR proteins whose expression was lost)	MSI status by Promega MSI Analysis System (number of markers out of 5 altered)
Endometrium	E1	70	2019	15	dMMR ( MLH1, PMS2)	MSI ( 5/5)
	E2	75	2018	20.1	dMMR ( MLH1, PMS2)	MSI ( 4/5)
	E3	60	2017	16	dMMR ( MLH1, PMS2)	MSI ( 5/5)
	E4	70	2019	6.8	dMMR ( MLH1, PMS2)	N/A
	E5	75	2018	23.6	dMMR ( MSH2, MSH6)	MSI ( 5/5)
	E6	50	2018	61	dMMR ( MSH2, MSH6)	MSI ( 5/5)
	E7	90	2017	66.7	dMMR ( MSH6)	MSI ( 5/5)
	E8	40	2013	53.1	dMMR ( MSH2)	MSI ( 5/5)
	E9	90	2017	72.9	dMMR ( PMS2)	MSS ( 0/5)
	E10	70	2013	30	Non-contributory ( low and heterogeneous expression of MSH6 protein)	MSS ( 0/5)
	E11	20	2017	21.8	pMMR	MSS ( 0/5)
	E12	80	2017	32.4	pMMR	MSS ( 0/5)
	E13	80	2016	21	pMMR	MSS ( 0/5)
	E14	90	2015	55.6	pMMR	MSS ( 0/5)
	E15	80	2015	35.4	pMMR	MSS ( 0/5)
Colon-rectum	C16	60	2018	26.2	dMMR ( MLH1, MSH2, MSH6, PMS2)	MSI ( 5/5)
	C17	40	2013	12.6	dMMR ( MLH1, PMS2)	MSI ( 2/5)
	C18	70	2018	64.7	dMMR ( MLH1, PMS2)	MSI ( 5/5)
	C19	35	2019	29.1	dMMR ( MLH1, PMS2)	MSI ( 5/5)
	C20	50	2018	13.4	dMMR ( MLH1, PMS2)	MSI ( 5/5)
	C21	80	2016	36.4	dMMR ( MSH2, MSH6)	MSI ( 5/5)
	C22	60	2016	21	dMMR ( PMS2)	MSI ( 5/5)
	C23	70	2018	38.8	N/A	MSI ( 5/5)
	C24	50	2019	45.4	Non-contributory ( low and heterogeneous expression of MLH1 protein)	MSS ( 0/5)
	C25	40	2018	7.3	pMMR	MSS ( 0/5)
	C26	40	2015	47.4	pMMR	MSS ( 0/5)
	C27	70	2017	35	pMMR	MSS ( 0/5)
	C28	20	2018	18.8	pMMR	MSS ( 0/5)
	C29	60	2020	20.9	pMMR	MSS ( 0/5)
	C30	20	2018	9.2	pMMR	MSS ( 0/5)

For each sample, tumour content, sampling date, DNA concentrations, MMR status by IHC and MSI status by Promega MSI Analysis System are described.

dMMR: deficient MMR; MSI: microsatellite instability; MSS: microsatellite stability; N/A: not available; pMMR: proficient MMR.

Among the 15 samples from the patients with colorectal cancers (C16–C30), tumour content was from 20 to 80% and DNA concentrations were from 7.3 to 64.7 ng/µL. Eight out of the 15 samples were found dMMR/MSI (C16–C23). Based on the IHC results, 1 sample (C16) showed a loss of the 4 tested MMR proteins, 4 samples (C17–C20) had a loss of MLH1 and PMS2 proteins, 1 sample (C21) was found with altered MSH2 and MSH6 proteins, 1 sample (C22) had an altered PMS2 protein. IHC results from 1 sample (C23) was not available. One sample (C24) had IHC non-contributory results (due to a low and heterogeneous expression of MLH1 protein) while MSS status was obtained by PCR-based assay. IHC and PCR testing suggested pMMR/MSS in the 6 last samples (C25–C30).

### Custom capture-based NGS

NGS libraries were prepared from 100 ng of extracted tumour DNA using a custom-designed capture-based “Solid Tumour Solution” kit (Sophia Genetics, Saint-Sulpice, Switzerland) that covers 51 cancer-associated genes (regions of clinical interest in *AKT1, ALK, ARID1A, BRAF, BRCA 1, BRCA 2, CDK4, CDKN2A, CTNNB1, DDR2, DICER1, EGFR, ERBB2, ERBB4, ESR1, FBXW7, FGFR1, FGFR2, FGFR3, FOXL2, GNA11, GNAQ, GNAS, H3F3A, H3F3B, HIST1H3B, HRAS, IDH1, IDH2, KIT, KMT2A, KMT2D, KRAS, MAP2K1, MAP2K2, MET, MTOR, MYOD1, NRAS, PDGFRA, PIK3CA, PTPN11, RAC1, RAF1, RET, ROS1, SF3B1, SMAD4, TERT, TGFBR2* and *TP53* genes, see details in Supplementary Table S4 online). Targeted sequencing was performed using the MiSeq instrument (Illumina, San Diego, CA, USA). Generated raw NGS data were analyzed using the Sophia DDM software v.5.5.11 (Sophia Genetics). Briefly, the bioinformatics pipeline consists in an alignment of the fastq files to generate bam files (hg19 reference genome), then a variant calling for the determination of SNV and indels. All results are finally available in the Sophia DDM software as variants and low-confidence variants. A minimum of 300 × read depth and 95% coverage were required for each sample tested by NGS. A MSI algorithm module provided by Sophia DDM software was used for reads analysis at 6 unique MS regions for which 3 were split into forward (FWD) and reverse (REV) strands (*BAT25_FWD, BAT25_REV, BAT26_REV, CAT25_REV, NR-21_FWD, NR-21_REV, NR-22_FWD, NR-22_REV* and *NR-27_REV*). For each locus of each sample, 2 distance scores were calculated by comparing the homopolymer length of the MS region to a run-specific average length profile and a global average profile (calculated on more than 400 clinical profiles) respectively. A minimum of 100 × depth at each locus was required, otherwise the distance score could not be calculated. Run-specific and global overall distance scores were then determined by summing the distance scores obtained for the 6 MS loci. Weight coefficients were used to balance the distance scores with the 6 loci above (coefficient of 0.5 if both forward and reverse strands were analyzed, coefficient of 1 if only reverse strand was analyzed). The tumours were classified into 3 different phenotypes based on the highest overall distance score: scores above 14 defined MSI-HC (microsatellite instability with high confidence) status, scores between 6 and 14 defined MSI-LC (microsatellite instability with low confidence) status and scores less than 6 defined MSS (microsatellite stability) status. Matched normal tissues were not required for the determination of MSI status by this custom NGS approach.

### Idylla MSI assay

The Idylla system (Biocartis NV, Mechelen, Belgium) is a fully-automated real-time PCR-based platform with microfluidics processing that requires only 2 min of handling time. The Idylla MSI assay consists of a single-use cartridge with all reagents on board designed for the analysis of 7 MS regions (*ACVR2A, BTBD7, DIDO1, MRE11, RYR3, SEC31A, SULF2*)^50^. Briefly, macrodissected FFPE tumour sample sections were directly loaded in the Idylla MSI test cartridge (Biocartis) and inserted in the instrument. The number of tumour tissue sections used depends on the tissue surface. A 5 µm-thick FFPE tissue section was used for tissue surface > 50 mm^2^ and a 10 µm-thick FFPE tissue section was used for tissue surface > 25 mm^2^. Up to 5 tissue sections were used for lower tissue surface. A minimum of 20% tumours cells were needed in the region macrodissected. Inside the cartridge, the sample was then homogenized and cells lysed using a combination of high intensity focused ultrasound, enzymatic and chemical digestion and heat. The nucleic acids were liberated, PCR-amplified and detected using a fluorophore-based system. After a 150-min run, a final report was automatically generated on the Idylla console. Paired normal tissues were not required for comparison. A MSI algorithm, elaborated from a training set of several thousands of MSI clinical samples, determined a MSI-score for each biomarker. MSI-scores range from 0 to 1 with a set cut-off of ≥ 0.5 for positive results. Idylla result was considered valid if valid amplified signals were obtained for at least 5 out of 7 biomarkers. The tumours were defined as having MSI-H (microsatellite instability with high confidence) if at least 2 of the 7 MSI markers were positive, and MSS (microsatellite stability) if it did not meet these criteria.

### Bio-Rad Droplet digital PCR MSI assay

The Bio-Rad ddPCR MSI assay is based on the analysis of 5 MS markers (BAT25, BAT26, NR21, NR24, Mono27) into 3 distinct assays (MSI MPX1, MSI MPX2, MSI MPX3). The ddPCR assays were run in duplicate and performed using the QX200 Droplet Digital PCR System (Bio-Rad, Hercules, CA, USA). For each reaction, the ddPCR mixture consisted of 1 × ddPCR Multiplex Supermix for probes (Bio-Rad), 1X primer–probe mix and 10 ng of extracted tumour DNA, in a total volume of 22 µl. Positive, negative and no-template (nuclease-free water) controls were systematically used for each experiment. The droplet generation was performed in the QX200 Droplet Generator (Bio-Rad) using 20 μl of the ddPCR mixture and 70 μl of the droplet generation oil (Bio-Rad). An average of 15,000 droplets were generated per well. In case of less than 10,000 droplets were generated per well, the sample was repeated. Droplets were transferred into a 96-well plate for the thermal cycling amplification and sealed using the PX1 PCR Plate Sealer (Bio-Rad). The PCR protocol on a C1000 Touch Thermal Cycler (Bio-Rad) was as follows: 37° for 30 min, 95 °C for 10 min followed by 40 cycles of denaturation at 94 °C for 30 s, 55 °C for 1 min, with a final 10 min at 98°. After PCR amplification, fluorescence signals were quantified by the QX200 Droplet Reader (Bio-Rad) and data were analyzed using the QuantaSoft software v.1.7.4 (Bio-Rad). Positive and negative controls served as a guide to call markers. Using the free-hand pencil graphic tool, the cluster at the bottom left of the x–y plot was designed as the negative population (black dots in Supplementary Fig. S1 online). Clusters located vertically (in blue) and horizontally (in green) from the negative cluster were identified as the mutant population. Clusters located diagonally from the negative cluster represented the wild-type population (in red). Tumours were characterized for MSI phenotype by analyzing the results for all 5 markers: MSI-H (microsatellite instability with high confidence) tumours were defined by 2 or more MS markers altered while MSS (microsatellite stability) tumours showed none or one of the MS markers altered. Matched normal tissues were not required for comparison.

### Statistical analysis

The standard routine care, IHC and Promega MSI Analysis System, was considered as the gold standard. Performance of the three molecular-based assays (Bio-Rad MSI ddPCR assay, Idylla MSI assay and custom NGS) was computed based on the MSI/dMMR phenotype for the 30 samples.

For each molecular-based assay, the sensitivity and specificity were calculated as following:

- Sensitivity (Se) = number of MSI phenotype according to the molecular-based assay out of the number of MSI according to the standard routine care.
- Specificity (Sp) = number of MSS phenotype according to the molecular-based assay out of the number of MSS according to the standard routine care.
- Overall agreement = number of concordant phenotype between the molecular-based assay and to standard routine care out of the overall number of samples.

The 95% confidence interval (95%CI) was computed with exact Clopper-Pearson method using SAS software, version 9.4 (SAS Institute Inc., Cary, NC, USA).

## Supplementary information

Supplementary Information.

## Data Availability

The data generated during the current study are available from the corresponding author on reasonable request.
