# Supplementary material for: Evaluation of 3 molecular-based assays for microsatellite instability detection in formalin-fixed tissues of patients with endometrial and colorectal cancers
Source: Sci Rep. 2020 Oct 2;10:16386. doi: 10.1038/s41598-020-73421-5 (PMC7532161; doi:10.1038/s41598-020-73421-5)

Evaluation of 3 molecular-based assays for microsatellite instability detection in formalin-fixed tissues of patients with endometrial and colorectal cancers

Pauline Gilson[1*], Julien Levy[2], Marie Rouyer[2], Jessica Demange[2], Marie Husson[2], Céline Bonnet[3], Julia Salleron[4], Agnès Leroux[2], Jean-Louis Merlin[1], Alexandre Harlé[1]

Supplementary Table S1. Tumour genetic mutations detected in the 30 samples by the custom 51-gene NGS panel.

Sample ID	Gene	Exon	Base change	Amino acid change	Read depth	Variant allele frequency [%]
E1	ESR1	8	c.1348T>G	p.(Ser450Ala)	2541	12.7
E2	ARID1A	20	c.5548delG	p.(Asp1850Thrfs*33)	1542	27.9
	TERT	promoter region	c.-146G>A	p.?	1019	24.7
E3	ARID1A	20	c.5548delG	p.(Asp1850Thrfs*33)	3640	15.2
	AKT1	3	c.49G>A	p.(Glu17Lys)	4847	47
	KRAS	2	c.38G>A	p.(Gly13Asp)	3466	17.5
E4	ARID1A	20	c.5548del	p.(Asp1850Thrfs*33)	1526	20.7
	PIK3CA	2	c.263G>A	p.(Arg88Gln)	2163	31.2
E5	ARID1A	20	c.5548dupG	p.(Asp1850Glyfs*4)	2020	40.2
	PIK3CA	2	c.112C>T	p.(Arg38Cys)	522	30.3
	PIK3CA	21	c.3062A>G	p.(Tyr1021Cys)	1115	36.8
E6	PIK3CA	10	c.1633G>A	p.(Glu545Lys)	1596	15
E7	FBXW7	8	c.1039C>T	p.(Arg347Cys)	152	25.7
E8	CTNNB1	3	c.97T>G	p.(Ser33Ala)	3720	29.7
	ESR1	10	c.1607T>C	p.(Leu536Pro)	3545	28.6
	PIK3CA	21	c.3140A>T	p.(His1047Leu)	3983	28.3
E9	AKT1	3	c.49G>A	p.(Glu17Lys)	301	44.7
E10	CTNNB1	3	c.94G>T	p.(Asp32Tyr)	2585	36.5
E11	CTNNB1	3	c.110C>T	p.(Ser37Phe)	3355	6.6
	AKT1	3	c.49G>A	p.(Glu17Lys)	3255	6.2
E12	KRAS	2	c.35G>T	p.(Gly12Val)	4361	59.1
E13	TP53	9	c.949del	p.(Gln317Serfs*28)	1652	21.1
E14	TP53	7	c.743G>A	p.(Arg248Gln)	2422	80.6
	FBXW7	9	c.1082G>T	p.(Arg361Leu)	5216	20.3
	PIK3CA	2	c.323G>A	p.(Arg108His)	10242	91.7
E15	PIK3CA	21	c.3140A>G	p.(His1047Arg)	5559	31.4
C16	KRAS	2	c.35G>A	p.(Gly12Asp)	2961	26.2
C17	KRAS	2	c.35G>T	p.(Gly12Val)	628	6.9
	PIK3CA	2	c.113G>A	p.(Arg38His)	313	7.5
	FBXW7	10	c.1424T>C	p.(Ile475Thr)	656	9.2
C18	BRAF	15	c.1799T>A	p.(Val600Glu)	4261	22.6
	FBXW7	8	c.1039C>T	p.(Arg347Cys)	3986	21.8
	BRCA2	10	c.1813dupA	p.(Ile605Asnfs*11)	3072	22.2
C19	PIK3CA	21	c.3140A>G	p.(His1047Arg)	2204	18.9
	TP53	4	c.216del	p.(Val73Trpfs*50)	1995	19.2
C20	BRCA2	15	c.7543dupA	p.(Thr2515Asnfs*24)	365	33.3
	FBXW7	8	c.1040G>A	p.(Arg347His)	296	32.1
	CDKN2A	1	c.103G>T	p.(Gly35Trp)	2983	7.2

C21	PIK3CA	10	c.1637A>G	p.(Gln546Arg)	397	22
	BRCA2	11	c.2835del	p.(Asp946Ilefs*14)	205	27.3
C22	BRAF	15	c.1799T>A	p.(Val600Glu)	543	28.2
C23	BRAF	15	c.1799T>A	p.(Val600Glu)	1757	16.2
	PIK3CA	21	c.3140A>G	p.(His1047Arg)	1738	17.9
	TP53	8	c.817C>T	p.(Arg273Cys)	1841	17.2
C24	TP53	5	c.405_406delCC	p.(Cys135*fs*1)	1921	48
C25	KRAS	2	c.35G>A	p.(Gly12Asp)	2952	30.7
	TP53	7	c.700T>C	p.(Tyr234His)	3233	55.1
C26	KRAS	2	c.34G>T	p.(Gly12Cys)	1811	57.8
	TP53	7	c.742C>T	p.(Arg248Trp)	3028	66
	SMAD4	11	c.1333C>T	p.(Arg445*)	767	7.8
C27	KRAS	2	c.35G>T	p.(Gly12Val)	3821	28.8
C28	KRAS	2	c.35G>C	p.(Gly12Ala)	2761	37.3
	TP53	5	c.524G>A	p.(Arg175His)	3489	40
C29	GNAS	8	c.2531G>A	p.(Arg844His)	7719	3.3
	KRAS	2	c.35G>T	p.(Gly12Val)	4689	39.5
	PIK3CA	21	c.3140A>G	p.(His1047Arg)	5124	33.9
C30	BRAF	11	c.1397G>A	p.(Gly466Glu)	2643	10.4
	NRAS	2	c.34G>A	p.(Gly12Ser)	4260	17.6
	TP53	7	c.725G>T	p.(Cys242Phe)	1770	14.9

Supplementary Table S2. Details of E8 and C27 MSI discordant results among capture-based NGS, Idylla MSI, Bio-Rad ddPCR MSI, IHC and Promega MSI Analysis approaches.

Abbreviations: dMMR: deficient MMR; FWD: forward; MSI: microsatellite unstable; MSI-HC: microsatellite unstable with high confidence, MSI-LC: microsatellite unstable with low confidence; MSS: microsatellite stable, N/A: not available; pMMR: proficient MMR; REV: reverse.

Sample ID	DNA quality (ΔCq)	IHC (MMR proteins whose expression was lost)	Promega MSI Analysis System (global MSI status; stability/unstability of the 5 markers analyzed)		Idylla MSI assay (global MSI status; score for each of the 7 markers analyzed)		Capture-based NGS (Run-specific / global overall distance scores; Run-specific / global distance scores for each of the 9 markers analyzed)		Bio-Rad ddPCR MSI assay
E8	4.1	dMMR (MSH2)	MSI		Invalid		MSS (1.6/1.2)		MSI
			BAT-25	*unstable*	*ACVR2A*	*Invalid*	*BAT25_FWD*	*0.1/0.2*	
			BAT-26	*unstable*	*BTBD7*	*0*	*BAT25_REV*	*0.1/0.1*	
			MONO-27	*unstable*	*DIDO1*	*0*	*BAT26_REV*	*0.5 /0.2*	
			NR-21	*unstable*	*MRE11*	*Invalid*	*CAT25_REV*	*0.3/0.1*	
			NR-24	*unstable*	*RYR3*	*0*	*NR21_ FWD*	*0.1/0.1*	
					SEC31A	*0*	*NR21_REV*	*0.1/0.1*	
					SULF2	*0*	*NR22_ FWD*	*0.1/0.1*	
							NR22_REV	*0.1/0.0*	
							NR27_REV	*0.2/0.1*	
C27	3.4	pMMR	MSS		MSS		MSI-LC (5.3/6.8)		MSS
			BAT-25	*stable*	*ACVR2A*	*0*	*BAT25_FWD*	*0.7/0.7*	
			BAT-26	*stable*	*BTBD7*	*0*	*BAT25_REV*	*0.7/0.8*	
			MONO-27	*stable*	*DIDO1*	*0*	*BAT26_REV*	*0.8 /1.6*	
			NR-21	*stable*	*MRE11*	*0*	*CAT25_REV*	*0.1/0.2*	
			NR-24	*stable*	*RYR3*	*0*	*NR21_ FWD*	*1.5/1.7*	
					SEC31A	*0*	*NR21_REV*	*1.4/1.5*	
					SULF2	*0*	*NR22_ FWD*	*0.0/0.1*	
							NR22_REV	*0.0/0.0*	
							NR27_REV	0.1/0.3	

Supplementary Table S3. Delay between the time of analysis and tumour sampling.

Sample ID	IHC and Promega MSI Analysis system		Idylla MSI assay		Custom capture-based NGS		Bio-Rad ddPCR MSI assay	
	[months]	[years]	[months]	[years]	[months]	[years]	[months]	[years]
**E1**	0	0	1	0	2	0	14	1
**E2**	0	0	0	0	1	0	14	1
**E3**	0	0	13	1	19	1	30	2
**E4**	0	0	2	0	12	1	14	1
**E5**	6	0	6	0	7	0	21	1
**E6**	0	0	5	0	1	0	13	1
**E7**	0	0	32	2	33	2	34	2
**E8**	34	2	76	6	75	6	77	6
**E9**	0	0	29	2	29	2	30	2
**E10**	62	5	66	5	62	5	74	6
**E11**	2	0	32	2	32	2	34	2
**E12**	0	0	33	2	32	2	34	2
**E13**	0	0	38	3	38	3	40	3
**E14**	0	0	50	4	49	4	51	4
**E15**	1	0	48	4	47	3	49	4
**C16**	12	1	4	0	3	0	13	1
**C17**	63	5	63	5	64	5	78	6
**C18**	0	0	1	0	5	0	17	1
**C19**	0	0	0	0	6	0	7	0
**C20**	0	0	15	1	14	1	16	1
**C21**	0	0	36	3	41	3	37	3
**C22**	0	0	41	3	46	3	42	3
**C23**	/	/	11	0	11	0	21	1
**C24**	1	0	2	0	2	0	10	0
**C25**	0	0	4	0	1	0	13	1
**C26**	0	0	42	3	39	3	50	4
**C27**	0	0	28	2	27	2	30	2
**C28**	0	0	17	1	16	1	18	1
**C29**	0	0	2	0	1	0	2	0
**C30**	12	0	24	2	24	2	25	2
**Median [inter-quartile range]**	**0** [0;1]	**0** [0;0]	**20.5** [4;37.5]	**1** [0;3]	**21.5** [5.3;38.8]	**1** [0;3]	**27.5** [14;39.3]	**2** [1;3]

Supplementary Table S4. Details of regions of interest in genes analysed by the custom NGS approach.

Gene	Reference sequences	Regions of interest
AKT1	NM_001014431.2	exon 3
ALK	NM_004304.5	exons 21-25
ARID1A	NM_006015.6	chr1:27056211-27056211_C_T ;<br>chr1:27106878-27106878__C ;<br>chr1:27105725-27105725_A_G ;<br>chr1:27106002-27106002_C_G ;<br>chr1:27023008-27023008__GGC ;<br>chr1:27087548-27087548_C_T ;<br>chr1:27100973-27100973_G_A ;<br>chr1:27102119-27102119_C_A;<br>chr1 :27099947-27099947_C_T ;<br>chr1:27105931-27105931_G_ ;<br>chr1:27106667-27106667__TT
BRAF	NM_004333.4	exons 11 and 15
BRCA1	NM_007294.3	exons 2-23
BRCA2	NM_000059.3	exons 2-27
CDK4	NM_000075.3	exon 2
CDKN2A	NM_000077.4	exons 1-3
CTNNB1	NM_001098209.2	exon 3
DDR2	NM_001014796.3	exon 18
DICER1	NM_001195573.1	exons 24 and 25
EGFR	NM_005228.5	exons 18-21
ERBB2	NM_001005862.2	exons 8, 17 and 20
ERBB4	NM_001042599.1	exons 10 and 12
ESR1	NM_001122742.1	exons 2-8 (excluding UTR3 regions)
FBXW7	NM_001013415.2	exons 8-12
FGFR1	NM_001174063.2	exons 13 and 15
FGFR2	NM_000141.5	exons 7, 12 and 14
FGFR3	NM_000142.4	exons 7, 9, 14 and 16
FOXL2	NM_023067.4	exon 1
GNA11	NM_002067.5	exons 4 and 5
GNAQ	NM_002072.5	exons 4 and 5
GNAS	NM_016592.4	exon 8
H3F3A	NM_002107.5	exon 2
H3F3B	NM_005324.5	exon 2
HIST1H3B	NM_003537.4	exon 1
HRAS	NM_001130442.2	exons 2-4
IDH1	NM_005896.3	exon 4
IDH2	NM_002168.4	exon 4
KIT	NM_000222.3	exons 8-11, 13, 17 and18
KMT2A	NM_001197104.2	chr11:118348829-118348829 ;<br>chr11:118342559-118342559
KMT2D	NM_003482.3	chr12:49431880-49431880 ;<br>chr12:49443567-49443567 ;<br>chr12:49425386-49425386 ;<br>chr12:49445095-49445095 ;<br>chr12:49436060-49436060
KRAS	NM_004985.5	exons 2-4

MAP2K1	NM_002755.4	exons 2 and 3
MAP2K2	NM_030662.4	exons 2-7
MET	NM_000245.4	exons 2 and 14-20
MTOR	NM_004958.4	exons 46-52
MYOD1	NM_002478.5	exon 1
NRAS	NM_002524.5	exons 2-4
PDGFRA	NM_006206.6	exons 12, 14 and 18
PIK3CA	NM_006218.4	exons 1, 2, 4, 5, 7, 9 and 20
PTPN11	NM_002834.5	exon 3
RAC1	NM_006908.5	exon 3
RAF1	NM_002880.3	exons 7, 10, 12, 13, 14 and 15
RET	NM_020630.5	exons 11, 13, 15 and 16
ROS1	NM_002944.2	exons 38 and 41
SF3B1	NM_012433.4	exons 15-17
SMAD4	NM_005359.6	exons 8-12
TERT	NM_001193376.2	Promoter regions and exons 1, 8, 9 and 13
TGFBR2	NM_001024847.2	chr3:30691872-30691872_A_ ;<br>chr3:30691872-30691872__AA ;<br>chr3:30732969-30732969_C_T ;<br>chr3:3071347830713481_CAGA_ ;<br>chr3:30732969-30732969_C_T ;<br>chr3:30729932-30729933_GA_ ;<br>chr3:3071359430713611_TTCCTGACGGCTGAGGAG_ ;<br>chr3 :30713724-30713724_ T_C
TP53	NM_000546.5	exons 2-11

Supplementary Figure S1. Examples of ddPCR result plots obtained for (**a**) E14 (MSS) and (**b**) C21 (MSI) samples using the QuantaSoft software v.1.7.4 (Bio-Rad).

The bottom left cluster (black dots) is defined as the negative population. Clusters located vertically (in blue) and horizontally (in green) from the negative cluster are identified as the mutant population. Clusters located diagonally from the negative cluster represented the wild-type population (in red).

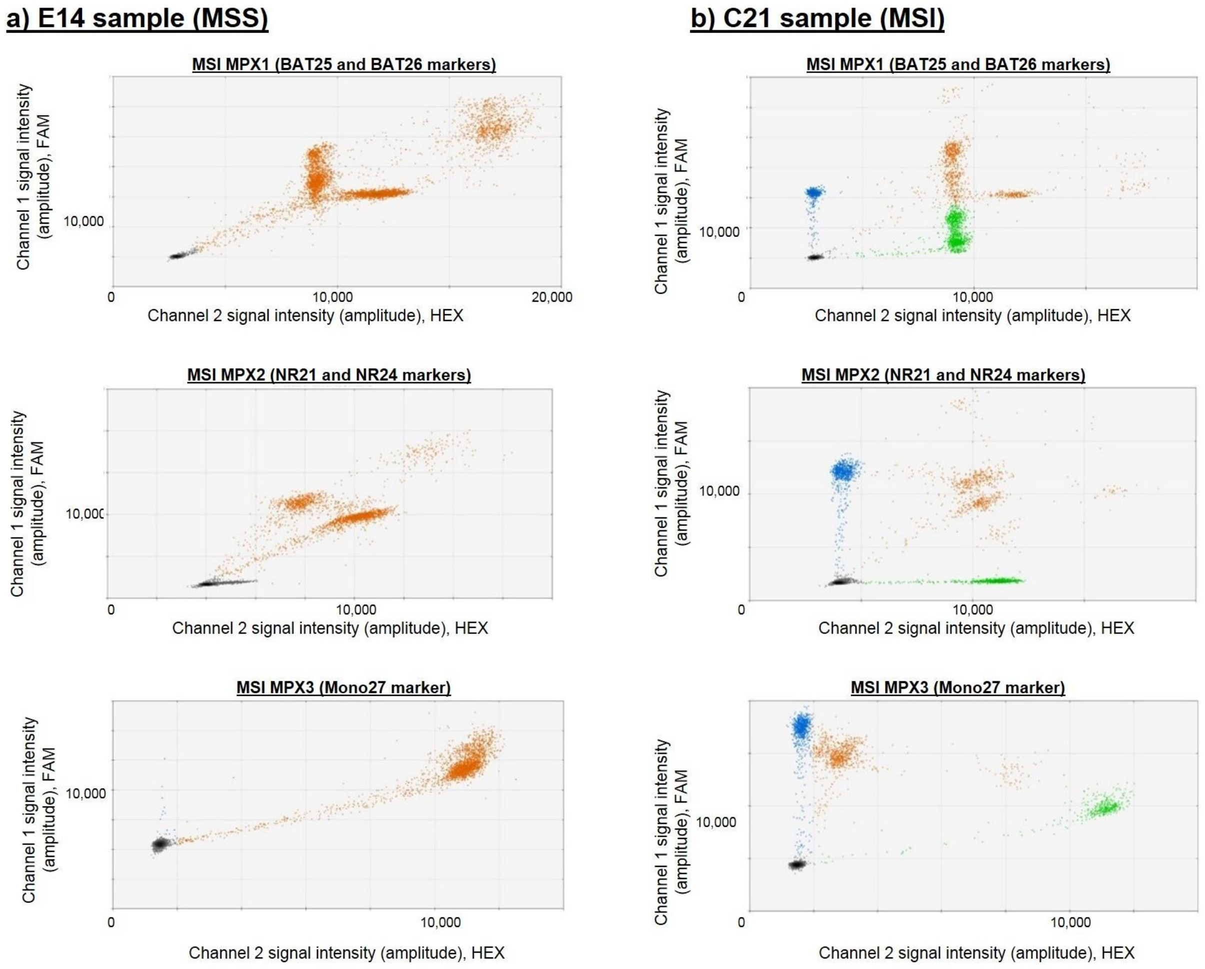

Supplement: Supplementary file 1 — Supplementary Information. [file 41598_2020_73421_MOESM1_ESM.pdf]
